# Therapeutic efficacy comparison of radiofrequency ablation in hepatocellular carcinoma and metastatic liver cancer

**DOI:** 10.3892/etm.2014.1505

**Published:** 2014-01-27

**Authors:** JING LIU, LIN-XUE QIAN

**Affiliations:** Department of Ultrasound, Beijing Friendship Hospital, Capital Medical University, Beijing 100050, P.R. China

**Keywords:** radiofrequency ablation, hepatocellular carcinoma, metastatic liver cancer, survival rate

## Abstract

The aim of this study was to evaluate the effect of radiofrequency ablation (RFA) on malignant hepatic tumors and compare its therapeutic efficacy in hepatocellular carcinoma (HCC) and metastatic liver cancer (MLC). A total of 56 patients with malignant hepatic tumors (34 patients with HCC and 22 patients with MLC) underwent RFA treatment. Two weeks following the RFA treatment, contrast-enhanced abdominal computed tomography scans were used to investigate whether the ablation of the tumors was complete. The patients were followed up for a period ranging from 1 to 93 months, to compare recurrence rates, distant recurrence rates and survival rates. The HCC group exhibited an initial complete ablation rate of 94.1% compared with 95.4% for the MLC group; the difference in ablation rates was not identified to be statistically significant. The recurrence and distant recurrence rates were 14.7% and 11.8%, respectively, for the HCC group and 9.1% and 36.4%, respectively, for the MLC group. The 1-, 3- and 5-year survival rates of the patients with HCC were 86.2, 71.4 and 60.0%, respectively, whereas those for the patients with MLC were 73.9%, 45.4% and 37.5%, respectively. The survival rates of the two groups were identified to be significantly different (P=0.002). RFA treatment was therefore shown to be effective in treating small (<5 cm) malignancies, which is clinically significant.

## Introduction

Liver cancer is one of the most common type of malignant tumor and is associated with a high mortality rate. Primary treatment methods include surgery, chemotherapy and ablation therapy. Radiofrequency ablation (RFA) therapy is considered to be a minimally invasive and localized treatment approach and its efficiency has been confirmed in a number of studies (1–3). With regard to early primary liver cancer, the effect of RFA treatment is comparable to that of surgical treatment (4–7). However, whether RFA is able to achieve the same therapeutic effect for metastatic liver cancer (MLC) tumors that are <5 cm in diameter is unknown. Therefore, the present study observed the treatment of patients with MLC and analyzed ~8 years of treatment data and the long-term follow-up of these patients.

## Materials and methods

### Patients and lesions

Between August 2005 and May 2013, 56 patients with liver cancer underwent ultrasound (US)-guided RFA in the Department of Ultrasound of Beijing Friendship Hospital (Beijing, China). The patients included: i) 34 cases of hepatocellular carcinoma (HCC) which consisted of 18 class-A, 14 class-B and two class-C cases, based on the 2010 American Association for the Study of Liver Diseases (AASLD) (8); ii) 22 cases of MLC which consisted of primary tumors located in the gastrointestinal tract (14 cases), kidney (two cases), lung (one case) and five cases from other locations. Diagnostic procedures were performed and included contrast-enhanced abdominal computed tomography (CT) scans, magnetic resonance imaging (MRI) and/or a liver tumor biopsy employing US guidance. There were 43 male and 13 female patients, aged between 25 and 83 years (average age, 56.3±6.5 years) with tumor sizes ranging from 1.2 to 4.7 cm (average size, 3.2±0.8 cm). The exclusion criteria were as follows: i) The patient suffered from severe jaundice or ascites; ii) the patient suffered from severe liver and kidney dysfunction; iii) huge liver cancer (>5 cm) or patients with diffuse liver cancer and iv) the patient suffered from coagulation dysfunction or severe bleeding tendencies. This study was conducted in accordance with the Declaration of Helsinki. Approval was obtained from the Ethics Committee of Beijing Friendship Hospital. All patients provided written informed consent for participation in this study.

### RFA procedure and treatment

A pre-operative US or CT scan was performed to determine the tumor location, size and number. RFA was performed using a multitined RITA^®^ StarBurst^®^ device (RITA Medical Systems, Inc., Mountain View, CA, USA). Following routine disinfection procedures and an intravenous general anesthetic (propofol), the US-guided radiofrequency needle (with numerous fine-needle electrodes capable of expanding between 2.0 and 5.0 cm in diameter) was applied to the surface of the tumor. Gradual expansion of the needle, according to the size of the tumor, occurred until a pre-defined temperature (105°C) was reached. Hyperechoic changes within the tumor and the surrounding tissue were observed after ~15–20 min at which point the electrode was removed, and then heat to seal the needle tract. For tumors <3 cm in diameter, a single needle puncture at a single point was sufficient. However, for a tumor with a diameter of >3 cm, the treatment was required to extend beyond the tumor foci and the surrounding 1.0–2.0 cm; therefore, multi-point puncturing was conducted in order to ensure complete tumor ablation.

### Evaluation of therapeutic effect

Two weeks following the RFA treatment, an enhanced CT scan review was carried out. If the images of the ablated lesions on the arterial and portal regions of the liver exhibited no significant increases, it was determined that complete ablation had been achieved. If the edges of ablated lesions exhibited abnormal enhancement and color Doppler US examination demonstrated partial blood flow of the ablated lesion, then incomplete ablation was diagnosed and further treatment was required. Following 1 month of treatment, regular blood tests (for detection of liver function and α-fetoproteins) and imaging studies (abdominal enhanced CT and/or US imaging) were performed as a basis for the evaluation of tumor development. Subsequent to complete ablation of the lesion, the re-emergence of the blood supply and contrast enhancement at the location of the original lesion was considered to indicate localized tumor recurrence. Abnormal enhancement showing the presence of lesions outside the region occupied by the primary tumors was considered to indicate distant tumor recurrence. The follow-up period ranged between 1 and 93 months.

### Statistical analysis

The 1-, 3- and 5-year survival rates of HCC and MLC following RFA treatment were calculated using the Kaplan-Meier method. The log-rank test was used to compare the survival curve differences between the HCC and the MLC groups. Furthermore, the differences in their complete ablation rates, recurrence rates and distant recurrence rates were evaluated with the χ^2^ test and continuity correction.

## Results

### Evaluation of therapeutic effect

**Figure 1:**
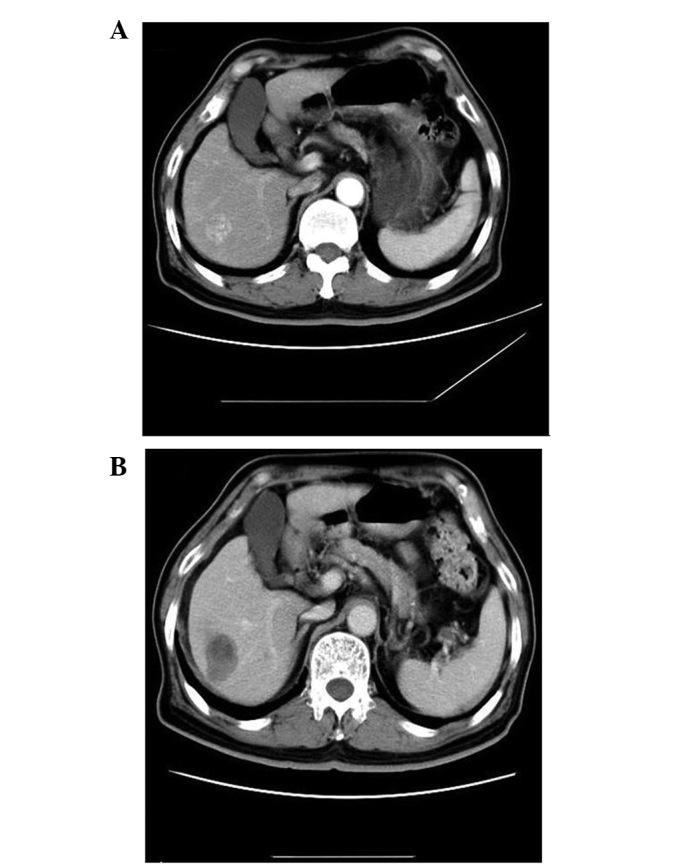
Comparison prior to and following tumor treatment. (A) Arterial region prior to treatment; the tumor was significantly enhanced. (B) Arterial region following the treatment; the tumor exhibited no obvious enhancement.

Two weeks following RFA treatment, enhanced CT scans were conducted to observe the treated tumors. In the HCC group (n=34), 32 patients exhibited complete ablation and in the MLC group (n=22), 21 patients exhibited complete ablation. Enhanced CT scans of these patients identified no significant enhancement in the arterial region (Fig. 1). The complete ablation rate was not identified to be statistically significant between the two groups (P=1.000). The patients were followed up for between 1 and 93 months. Five cases in the HCC group (5/34, 14.7%) showed cancer recurrence and four cases (11.8%) exhibited distant recurrence; in the MLC group, two cases (9.1%) showed cancer recurrence and eight cases (36.4%) exhibited distant recurrence (Table I). The difference between the two groups was not identified to be significant. The 1-, 3- and 5-year survival rates of HCC and MLC groups as of May 2013 are shown in Table II.

### Survival rate comparison between the HCC and MLC groups

**Figure 2:**
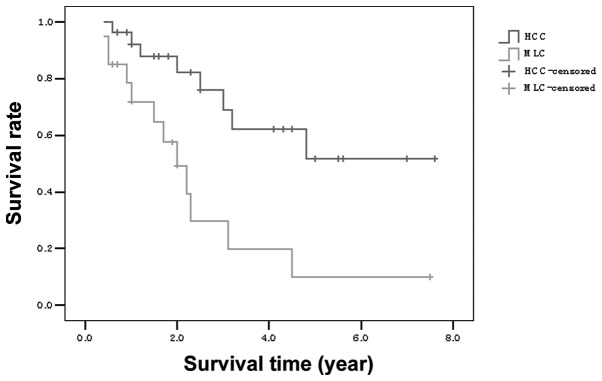
Survival curves for patients with HCC and MLC. HCC, hepatocellular carcinoma; MLC, metastatic liver cancer.

The 1-, 3- and 5-year survival rates were identified to be significantly different between the two groups (P=0.002; Fig. 2).

### Adverse effects

Following RFA treatment of the 48 patients, one patient experienced hepatic subcapsular hematoma; however, injection of prothrombin complex promoted hemostasis and the bleeding stopped. Three patients exhibited abnormal liver function following treatment, which was demonstrated by increased levels of alanine aminotransferase (ALT) and aspartate aminotransferase (AST). Diammonium glycyrrhizinate (a hepatoprotective drug) was administered, resulting in a gradual return of ALT and AST to normal levels. One patient developed a secondary infection, the symptoms of which were eased by performing a US-guided percutaneous catheter drainage. The remaining patients were closely observed following RFA treatment, and no skin burns, bleeding, nausea, vomiting or other symptoms were observed.

## Discussion

Numerous clinical studies have demonstrated that RFA is an effective treatment for focal liver tumors. In the present study, the complete ablation rate, following initial RFA treatment, in the HCC and MLC groups was 94.1% (32/34) and 95.4% (21/22), respectively. The initial complete ablation rate was predominantly related to tumor size; for small HCCs (≤3 cm), RFA has been found to be an effective and safe first-line treatment (9,10). In addition, the tumor location influenced the efficacy of RFA; the treatment effect is generally poor on tumors which neighboring the portal vein, diaphragm, gall bladder and bowel (11–14).

Surgical resection is often the preferred treatment for HCC and MLC as the patient’s 5-year survival rate is >50% (7). However, surgical resections should not be performed on patients suffering from multiple diseases, anatomical constraints, lack of hepatic functional reserve and extrahepatic metastasis, amongst other complications; RFA is therefore used as an alternative treatment. The development of imaging techniques has enabled the early diagnosis of liver lesions. Previous clinical studies have identified that RFA and surgical resection have similar efficacies in the treatment of small HCC; however, RFA treatment requires a shorter hospital stay with fewer complications when compared with surgical resection (15,16). Peng *et al* (17) analyzed 145 patients with small HCCs; 74 patients received a surgical resection, while 71 patients underwent RFA treatment. The study revealed that patients who underwent RFA treatment exhibited 1-, 3- and 5- year survival rates of 98.5, 87.7 and 71.9%, respectively, compared with 90.5, 70.9 and 62.1% for the patients who underwent resection. This was considered to be due to the trauma, various complications and significant side-effects associated with the surgery.

In the current study, following RFA treatment the patients with HCC exhibited 1-, 3- and 5- year survival rates of 86.2, 71.4 and 60.0%, demonstrating that RFA is comparable with surgical resection in the effective treatment of small HCC (<5 cm) tumors. Patients with MLC exhibited 1-, 3- and 5- year survival rates of 73.9, 45.4 and 37.5%; the survival rates of the two groups were significantly different. Veltri *et al* (6) observed that the survival rates 1, 3 and 5 years after RFA treatment were 84, 43 and 23% in patients with liver metastases resulting from colorectal cancer. In the previous study, the mortality of one MLC patient was observed to be due to liver failure, hepatorenal syndrome and hepatic encephalopathy and there were three fatalities as a result of multiple organ metastases. The majority of patients receiving MLC treatment have advanced stage cancer; therefore, the application of RFA treatment in these cases avoids the trauma of surgery, alleviates suffering and prolongs patient survival to a certain extent (18,19).

In conclusion, RFA is a minimally invasive technique that is economical, highly accurate, safe, effective, reliable and reproducible. The reduced postoperative complications improve patient quality of life and prolong lifespan; therefore, this method may be beneficial if applied in clinical treatment.

## Figures and Tables

**Table I:** Local therapeutic efficacy comparison of the HCC group (n=34) and the MLC group (n=22) following RFA.

Group	Complete ablation rate (%)	Recurrence rate (%)	Distant recurrence rate (%)
HCC	32/34 (94.1)	5/34 (14.7)	4/34 (11.8)
MLC	21/22 (95.4)	2/22 (9.1)	8/22 (36.4)
P-value	1.000	0.836	0.063

HCC, hepatocellular carcinoma; MLC, metastatic liver cancer; RFA, radiofrequency ablation.

**Table II:** Survival rate of 56 patients following RFA treatment.

	Survival rate (%)		
	
Group	1 year	2 year	3 year
HCC	86.2	71.4	60.0
MLC	73.9	45.4	37.5
Total	80.9	60.0	50.0

HCC, hepatocellular carcinoma; MLC, metastatic liver cancer; RFA, radiofrequency ablation.

## References

[1] Ng KK, Poon RT, Lo CM, Yuen J, Tso WK, Fan ST (2008). Analysis of recurrence pattern and its influence on survival outcome after radiofrequency ablation of hepatocellular carcinoma. J Gastrointest Surg.

[2] Kudo M (2010). Radiofrequency ablation for hepatocellular carcinoma: updated review in 2010. Oncology.

[3] Tiong L, Maddern GJ (2011). Systematic review and meta-analysis of survival and disease recurrence after radiofrequency ablation for hepatocellular carcinoma. Br J Surg.

[4] Livraghi T, Meloni F, Di Stasi M (2008). Sustained complete response and complications rates after radiofrequency ablation of very early hepatocellular carcinoma in cirrhosis: Is resection still the treatment of choice?. Hepatology.

[5] Naugler WE, Sonnenberg A (2010). Survival and cost-effectiveness analysis of competing strategies in the management of small hepatocellular carcinoma. Liver Transpl.

[6] Veltri A, Guarnieri T, Gazzera C (2012). Long-term outcome of radiofrequency thermal ablation (RFA) of liver metastases from colorectal cancer (CRC): size as the leading prognostic factor for survival. Radiol Med.

[7] Tombesi P, Di Vece F, Sartori S (2013). Resection vs thermal ablation of small hepatocellular carcinoma: What’s the first choice?. World J Radiol.

[8] Bruix J, Sherman M, American Association for the Study of Liver Diseases (2011). Management of hepatocellular carcinoma: an update. Hepatology.

[9] Choi D, Lim HK, Rhim H (2007). Percutaneous radiofrequency ablation for early-stage hepatocellular carcinoma as a first-line treatment: long-term results and prognostic factors in a large single-institution series. Eur Radiol.

[10] Kim YS, Lim HK, Rhim H (2013). Ten-year outcomes of percutaneous radiofrequency ablation as first-line therapy of early hepatocellular carcinoma: analysis of prognostic factors. J Hepatol.

[11] Kariyama K, Nouso K, Wakuta A (2011). Percutaneous radiofrequency ablation for treatment of hepatocellular carcinoma in the caudate lobe. AJR Am J Roentgenol.

[12] Tang Z, Fang H, Kang M (2011). Percutaneous radiofrequency ablation for liver tumors: Is it safer and more effective in low-risk areas than in high-risk areas?. Hepatol Res.

[13] Huang HW (2013). Influence of blood vessel on the thermal lesion formation during radiofrequency ablation for liver tumors. Med Phys.

[14] Morimoto M, Numata K, Kondo M (2013). Radiofrequency ablation combined with transarterial chemoembolization for subcapsular hepatocellular carcinoma: a prospective cohort study. Eur J Radiol.

[15] Sato M, Tateishi R, Yasunaga H (2012). Mortality and morbidity of hepatectomy, radiofrequency ablation, and embolization for hepatocellular carcinoma: a national survey of 54,145 patients. J Gastroenterol.

[16] Guo WX, Sun JX, Cheng YQ (2013). Percutaneous radiofrequency ablation versus partial hepatectomy for small centrally located hepatocellular carcinoma. World J Surg.

[17] Peng ZW, Lin XJ, Zhang YJ (2012). Radiofrequency ablation versus hepatic resection for the treatment of hepatocellular carcinomas 2 cm or smaller: a retrospective comparative study. Radiology.

[18] Stang A, Fischbach R, Teichmann W, Bokemeyer C, Braumann D (2009). A systematic review on the clinical benefit and role of radiofrequency ablation as treatment of colorectal liver metastases. Eur J Cancer.

[19] Wong SL, Mangu PB, Choti MA (2010). American Society of Clinical Oncology 2009 clinical evidence review on radiofrequency ablation of hepatic metastases from colorectal cancer. J Clin Oncol.

